# Epigenetic loci for blood pressure are associated with hypertensive target organ damage in older African Americans from the genetic epidemiology network of Arteriopathy (GENOA) study

**DOI:** 10.1186/s12920-020-00791-0

**Published:** 2020-09-11

**Authors:** Minjung Kho, Wei Zhao, Scott M. Ratliff, Farah Ammous, Thomas H. Mosley, Lulu Shang, Sharon L. R. Kardia, Xiang Zhou, Jennifer A. Smith

**Affiliations:** 1 Department of Epidemiology, School of Public Health, University of Michigan, Ann Arbor, MI 48109 USA; 2 Memory Impairment and Neurodegenerative Dementia (MIND) Center, University of Mississippi Medical Center, Jackson, MS 39216 USA; 3 Department of Biostatistics, School of Public Health, University of Michigan, Ann Arbor, MI 48109 USA

**Keywords:** Target organ damage, DNA methylation, Hypertension, Estimated glomerular filtration rate, Urinary albumin-creatinine ratio, Left ventricular mass, Relative wall thickness

## Abstract

**Background:**

Hypertension is a major modifiable risk factor for arteriosclerosis that can lead to target organ damage (TOD) of heart, kidneys, and peripheral arteries. A recent epigenome-wide association study for blood pressure (BP) identified 13 CpG sites, but it is not known whether DNA methylation at these sites is also associated with TOD.

**Methods:**

In 1218 African Americans from the Genetic Epidemiology Network of Arteriopathy (GENOA) study, a cohort of hypertensive sibships, we evaluated the associations between methylation at these 13 CpG sites measured in peripheral blood leukocytes and five TOD traits assessed approximately 5 years later.

**Results:**

Ten significant associations were found after adjustment for age, sex, blood cell counts, time difference between CpG and TOD measurement, and 10 genetic principal components (FDR q < 0.1): two with estimated glomerular filtration rate (eGFR, cg06690548, cg10601624), six with urinary albumin-to-creatinine ratio (UACR, cg16246545, cg14476101, cg19693031, cg06690548, cg00574958, cg22304262), and two with left ventricular mass indexed to height (LVMI, cg19693031, cg00574958). All associations with eGFR and four associations with UACR remained significant after further adjustment for body mass index (BMI), smoking status, and diabetes. We also found significant interactions between cg06690548 and BMI on UACR, and between 3 CpG sites (cg19693031, cg14476101, and cg06690548) and diabetes on UACR (FDR q < 0.1). Mediation analysis showed that 4.7% to 38.1% of the relationship between two CpG sites (cg19693031 and cg00574958) and two TOD measures (UACR and LVMI) was mediated by blood pressure (Bonferroni-corrected *P* < 0.05). Mendelian randomization analysis suggests that methylation at two sites (cg16246545 and cg14476101) in *PHGDH* may causally influence UACR.

**Conclusions:**

In conclusion, we found compelling evidence for associations between arteriosclerotic traits of kidney and heart and previously identified blood pressure-associated DNA methylation sites. This study may lend insight into the role of DNA methylation in pathological mechanisms underlying target organ damage from hypertension.

## Background

More than 100 million Americans have high blood pressure, which costs more than 50 billion dollars a year [1]. Hypertension is major modifiable risk factor for atherosclerosis and arteriosclerosis, the buildup of fatty deposits, inflammation, and scar tissue within the walls of arteries and arterioles, which can lead to target organ damage (TOD) of heart, kidneys, and peripheral arteries [2, 3]. TOD may act as a subclinical endpoint helping quantify total cardiovascular risk and informing treatment strategy [4, 5]. Since not all individuals with hypertension develop TOD, it is important to identify factors determining propensity for TOD including both environmental or genetic factors [6, 7].

Genome-wide association studies have identified genetic variation that influences TOD traits [8–13]. Epigenome-wide association studies (EWAS) are now beginning to identify epigenetic modifications that are associated with TOD [14], but the number of studies in this area is limited. DNA methylation of cytosine bases in CpG dinucelotides has gained attention as a new biomarker that may help shed light on novel mechanistic pathways in the etiology of complex traits and diseases, and may be promising intervention targets. Recently, a meta-EWAS of blood pressure conducted in populations of multiple ancestries (*N* = 17,010) identified 13 significant CpG sites [15]. In a cross-sectional study, two of the 13 CpG sites were also associated with blood pressure-determining hemodynamic factors including stroke volume and total peripheral resistance in 135 adolescents and 110 adults [16]. However, it is not known whether these 13 sites are also associated with TOD. Since only a limited number of risk factors have been identified for TOD, examining the association of the epigenetic pathways involved in the relationship between blood pressure and TOD may reveal new risk biomarkers for arteriosclerotic diseases. In addition, examining how those methylation changes are associated with changes in proximal gene expression might help us to better understand a mechanistic pathway by which methylation can influence TOD etiology.

Here, we investigate the association between the 13 DNA methylation sites associated with blood pressure and TOD of the heart, kidneys, and peripheral arteries in a cohort of largely hypertensive African Americans, and investigate whether these effects are modified by other risk factors for arteriosclerosis including body mass index (BMI), smoking status, and diabetes or are mediated by blood pressure.

## Methods

### Subjects

The Genetic Epidemiology Network of Arteriopathy (GENOA) is a community-based study designed to identify genes influencing blood pressure [17]. In the first phase of GENOA (Phase 1: 1996–2001), sibships with at least two adults with clinically diagnosed essential hypertension before age 60 were recruited, and all siblings in the sibship were invited to participate regardless of hypertension status [18]. In Phase 1, a total of 1583 European Americans from Rochester, MN and 1854 African Americans from Jackson, MS were enrolled. In the first follow-up phase of GENOA (Phase 2: 2000–2004), 1239 European American and 1482 African American participants were successfully re-recruited. The Phase 2 examination included measures of target organ damage to the heart, brain, kidneys, and peripheral arteries. DNA methylation profiles for 1416 African American participants were measured in whole blood samples collected at Phase 1. After exclusions for missing TOD or covariate data, a total of 1218 participants were included in the current study.

### Methylation measures

DNA methylation was assessed, and bisulfite converted from genomic DNA extracted from stored peripheral blood leukocytes collected at GENOA Phase 1 using Illumina Infinium HumanMethylation BeadChips (*N* = 316 from 450 K and *N* = 1100 from EPIC). The Minfi R package [19] was used to preprocess and normalize the data. The exclusion criteria for probes and software used are described in detail elsewhere [20]. Houseman’s method was used to estimate the proportion of each white blood cell type [21]. Methylation at each CpG site was summarized as an M-value, the log_2_ ratio of the intensity of the methylated probe versus the unmethylated probe [22]. M-values were first adjusted for batch effects using *ComBat* in R [23], and then adjusted for white blood cell proportions using linear regression prior to analysis. Thirteen CpG sites identified from a blood pressure meta-EWAS [15] were selected for further analysis.

### Gene expression measures

Gene expression levels in GENOA African Americans were measured in lymphoblastoid cell lines using the Affymetrix Human Transcriptome Array 2.0. All array images passed visual inspection using the Affymetrix Expression Console. A total of 1205 samples remained after quality control. Affymetrix CEL files were normalized using the Robust Multichip Average algorithm using Affymetrix Power Tool software [24]. The Brainarray custom CDF version 19 was used to map the probes to genes [25] and *Combat* in R was used to adjust batch effects and other technical covariates. A total of 32,955 autosomal gene expression measures were available after quantile normalization across genes.

### Genotyping and imputation for Mendelian randomization (MR) analysis

GENOA African American samples were genotyped on the Affymetrix® Genome-Wide Human SNP Array 6.0 platform, Illumina® Human1M-Duo, or Human660W-Quad BeadChips. Participants were excluded if they had an overall SNP call rate < 95% or sex mismatch between genotypic and phenotypic measurement. SNPs were excluded if they had unknown chromosomal location, < 95% call rate, or minor allele frequency (MAF) less than 0.01. Imputation was performed separately by chip (Affymetrix or Illumina) using the 1000G Phase3 v5 reference panel, and post-imputation comparison between the two groups revealed no substantial differences in genotype and allele frequencies. SNPs with imputation quality less than 0.8 were removed before MR analysis.

### Clinical assessments and covariate definitions

Measures of systolic blood pressure (SBP) and diastolic blood pressure (DBP) were the average of the last two of three sequential readings taken in a sitting position for at least 5 min at Phase 1 [18]. SBP and DBP were adjusted for anti-hypersensitive medication by adding 15 and 10 mmHg, respectively [26]. TOD measures collected at Phase 2 included two kidney measures (estimated glomerular filtration rate, eGFR; urinary albumin-to-creatinine ratio, UACR), two echocardiographic measures of the heart (relative wall thickness, RWT; left ventricular mass indexed to height^2.7^, LVMI), and a measure of the peripheral arteries (ankle-brachial index, ABI). TOD measurement methods are described in detail elsewhere [20]. BMI was calculated as weight (kg) divided by the square of height (m). Smoking status was characterized as current or non-smoker. Diabetes was defined as fasting serum glucose concentration ≥ 126 mg/dl or self-reported physician-diagnosed diabetes and current diabetes medication use.

### Statistical analysis

Outliers greater than four standard deviations from the mean of each TOD outcome and covariate were removed prior to analysis, and UACR was natural log transformed as ln (UACR+ 1). Ten genotype principal components (PCs) were included in all models to adjust for population stratification. Analyses were conducted in R (Version 3.4.1) [27] and SAS 9.4 (SAS Institute, Cary NC). To examine the effect size of CpG sites on blood pressure and TOD traits comparably, we used methylation M-values as the independent variable in both association analyses and used blood pressure/TOD traits as the dependent variable.

#### CpG associations with blood pressure

In the Richard et al. (2017) EWAS, a subset of GENOA African Americans with 450 K methylation data (*N* = 422) were included. To examine whether the blood pressure-associated CpG sites from Richard et al. (2017) were also associated with SBP and DBP in the full GENOA sample with methylation data and TOD traits (*N* = 1212), we used a linear mixed effects model while accounting for family structure. Covariates included age at Phase 1, sex, BMI, smoking status, and 10 PCs.

#### CpG associations with TOD and interaction with TOD risk factors

A linear mixed effects model accounting for family structure based on pedigree information was used to examine the association of the 13 CpG sites with the five TOD measures in GENOA. In the baseline model, covariates included sex, age at Phase 1, age difference between Phase 1 and Phase 2, and 10 PCs. We further added traditional risk factors for arteriosclerosis including BMI, smoking status, or diabetes, respectively, into the models to examine their confounding or mediating effects. For CpG sites that had significant association with a TOD measure (false discovery rate, FDR q < 0.1), we tested for interaction between the CpG site and each of the three risk factors (BMI, smoking status, and diabetes) on the TOD measure. For each significant interaction, we plotted an interaction using a scatter plot by risk factor status (e.g. diabetes VS non-diabetes). The y-axis of each plot was a predicted value of TOD measure estimated from each linear regression model.

#### Mediation of CpG-TOD associations by blood pressure

For significant associations between CpG sites and TOD measures prior to adding arteriosclerosis risk factors (FDR q < 0.1), formal mediation analyses were conducted using the *mediation* R package to examine whether the effects of the CpG sites on TOD measures are mediated through blood pressure [28]. We used a linear mixed effect model accounting for family structure and adjusting for age at Phase 1 and sex. The formal mediation framework assumes no unmeasured confounding or effect modification between the included elements. In secondary analysis, we further included BMI, smoking status, and diabetes in the model because they are potential confounders and/or mediators in the mediating pathway we tested. The point estimates and 95% confidence intervals of indirect, direct, and total effects were obtained using bootstrapping with 100,00 iterations. The proportion mediated describes the average magnitude of the indirect association between a CpG site and a TOD measure attributed to changes in blood pressure (SBP or DBP) relative to the average total association.

#### Mediation of CpG-TOD associations by gene expression levels

To examine whether the associations between CpG sites and TOD measures prior to adding arteriosclerosis risk factors (FDR q < 0.1) are mediated by gene expression levels, we evaluated the associations between 1) CpG sites and gene expression levels from genes that were located within ±1 Mb of the corresponding CpG sites, and 2) between the gene expression levels and TOD traits, separately. For gene expression levels that had at least a marginal association (*P* < 0.1) both with a CpG site and a TOD measure, we performed a formal mediation analysis. We used a linear mixed effects model adjusted for family structure with covariates including sex, age when gene expression was measured, and 10 PCs. When DNA methylation M-value or gene expression level was the independent variable, it was pre-adjusted for sex and age before conducting the association analysis. In formal mediation analysis, we tested the indirect association between a CpG site and a TOD measure through changes in proximal gene expression levels using the bootstrapping method in the *mediation* R package [28].

#### Mendelian randomization analysis of CpG-TOD associations

To investigate whether the previously identified CpG sites (FDR q < 0.1 prior to adding arteriosclerosis risk factors) might contribute causally to TOD measures, we performed Mendelian randomization (MR) analysis using 1000G imputed SNPs in GENOA African Americans. Since public mQTL databases and TOD GWAS data are limited in African Americans, we performed one-sample MR analysis using data from GENOA African Americans. Instrumental variables (IVs) for DNA methylation were drawn from cis-mQTL analysis in GENOA using SNPs within ±1 Mb of each corresponding CpG site using RVTEST (*N* = 1151 and FDR q < 0.05) [29] and clumped for independence (r^2^ = 0.1 and physical distance threshold = 100 kb). The associations between IVs with CpGs and TOD measures were examined to obtain the beta and standard error estimates in GENOA African Americans. Inverse-variance weighting (IVW) was used to evaluate causation for each CpG on TOD measures. If the IV is comprised of only one variant, the Wald-ratio MR method was used to examine the causal effect [30]. Causal estimates were obtained using the *TwoSampleMR* R package [31]. We considered the Bonferroni corrected *P*-value to be significent (*P* < 0.005). For each significant MR result, we created a scatter plot to depict the relationship of the SNP effects on the exposure against the SNP effects on the outcome using the same package. A line was drawn in each plot and the slope corresponds to the estimated causal effect from the IVW method. We used the MR Egger test to examine pleiotropy because IVW MR is invalid in the presence of horizontal pleiotropic effects of IVs [32].

## Results

Descriptive statistics at Phase 1 (SBP and DBP) and Phase 2 (other variables) are shown in Table 1 for the 1218 GENOA participants. The percentage of females was slightly over 70%. The mean age was 63.6 years at Phase 2, with an average of 5.1 years between Phase 1 (methylation measurement) and 2 (TOD measurement). Eighty percent of participants were hypertensive, and their blood pressures were an average of 145.9 and 82.9 mmHg for SBP and DBP, respectively, after adjustment for taking anti-hypertensive medications. BMI was 31.7 kg/m^2^ (SD: 6.7), 12% of participants were current smokers, and 31% had diabetes.

**Table 1:** Characteristics of participants (*N* = 1218)

Variable	Mean (SD) or %
Female	71.4 (%)
Age (years)	63.6 (9.2)
Difference between Phases 1 and 2 (years)	5.1 (1.3)
BMI (kg/m^2^)	31.7 (6.7)
Educational attainment (years)	12.1 (3.7)
SBP (mmHg)^a^	145.9 (22.0)
DBP (mmHg)^a^	82.9 (11.1)
Current smoking	12.1 (%)
Hypertension	80.0 (%)
Taking anti-hypertensive medication	71.6 (%)
Diabetes	31.1 (%)
Estimated glomerular filtration rate, ml/min/1.73 m2 (*N* = 1212)	88.5 (21.1)
Urine albumin-to-creatinine ratio (*N* = 1214)^b^	51.5 (1.5)
Relative wall thickness (*N* = 1181)	0.3 (0.1)
Left ventricular mass index (g/height^2.7^) (*N* = 1171)	39.2 (10.1)
Ankle-brachial index (*N* = 1176)	1.0 (0.1)

SD: Standard Deviation, SBP: Systolic blood pressure, DBP: Diastolic blood pressure, Hypertension: systolic blood pressure ≥ 140, or diastolic blood pressure ≥ 90, or taking anti-hypertensive medication, Diabetes: sugar glucose level ≥ 126 or taking diabetes medication

^a^SBP and DBP at Phase 1. For participants taking anti-hypertensive medication, 15 and 10 mmHg were added to SBP and DBP, respectively

^b^Urine albumin-to-creatinine ratio was log transformed prior to analysis

Three and two out of the 13 evaluated CpG sites had at a least marginal association (*P* < 0.05) with SBP and DBP, respectively, after adjustment for age, sex, BMI, and smoking status: cg19693031 and cg00574958 with both SBP and DBP; cg18120259 with SBP only (Table 2). Three of the associations remained significant after Bonferroni correction for multiple testing (*P* < 0.0038) (cg19693031 with DBP, cg18120259 and cg00574958 with SBP). Effect directions for nine CpG sites for SBP and 12 CpG sites for DBP were negative in our analysis, while the effect directions for all of the 13 CpG sites were negative in Richard et al. (2017).

**Table 2:** Associations between CpG sites and blood pressure in Richard et al. (2017) and GENOA African Americans

				SBP				DBP			
				Richard et al.		GENOA (*N* = 1212)		Richard et al.		GENOA (*N* = 1212)	
CpG site	Chr	Position	Gene	Beta	P	Beta	P	Beta	P	Beta	P
cg23999170	1	115,628,111	TSPAN2	−0.0001	1.50E-10	−1.0749	0.4771	−0.0002	1.90E-13	− 1.3116	0.1148
cg16246545	1	120,255,941	PHGDH	−0.0002	1.20E-22	0.2953	0.8853	−0.0002	1.10E-09	0.7000	0.5343
cg14476101	1	120,255,992	PHGDH	− 0.0003	2.70E-34	−1.0872	0.4582	−0.0004	2.10E-21	−0.1893	0.8144
cg19693031	1	145,441,552	TXNIP	−0.0002	3.10E-29	−2.6897	0.0174	−0.0003	1.80E-14	−2.1234	**0.0006**
cg08035323	2	9,843,525	–	−0.0001	9.60E-07	0.4203	0.7385	−0.0003	2.60E-11	−0.6774	0.3283
cg06690548	4	139,162,808	SLC7A11	−0.0002	1.60E-32	0.2335	0.8484	−0.0003	7.90E-26	−0.2531	0.7062
cg18120259	6	43,894,639	LOC100132354	−0.0002	2.20E-21	−7.3227	**0.0038**	−0.0002	8.90E-14	−1.6515	0.2359
cg00533891	10	80,919,242	ZMIZ1	−0.0001	5.50E-09	−0.9521	0.5078	−0.0002	2.00E-11	−1.0884	0.1688
cg17061862	11	9,590,431	–	−0.0001	9.40E-12	−0.0880	0.9617	−0.0003	4.30E-13	−1.4197	0.1593
cg00574958	11	68,607,622	CPT1A	−0.0001	1.20E-13	−4.5052	**0.0008**	−0.0001	3.00E-10	−2.1141	0.0044
cg10601624	12	6,404,377	–	−0.0001	2.40E-16	1.2257	0.5309	−0.0002	4.30E-13	0.1518	0.8878
cg22304262	19	47,287,778	SLC1A5	−0.0001	1.40E-17	−1.4197	0.4095	−0.0002	9.60E-11	−1.6601	0.0796
cg02711608	19	47,287,964	SLC1A5	−0.0001	2.00E-21	0.3158	0.8198	−0.0002	4.30E-10	−0.2109	0.7822

N: sample size, Position: position at Hg19, SBP: Systolic blood pressure, DBP: Diastolic blood pressure, Chr: chromosome, Beta: beta coefficient, CpG: Cytosine-phosphate-guanine

Gene names are from UCSC Genome Browser

Coefficients from Richard et al. (2017) give the percent change in DNA methylation (beta-value) for every 1 mmHg change in blood pressure

Coefficients from GENOA give the change in blood pressure for every 1 change in DNA methylation (M-value)

All models were adjusted for age, sex, blood cell counts, BMI, smoking (current/former/never), and genetic principal components

In GENOA, *P*-value was bolded if *P* < 0.0038 (Bonferroni-adjusted alpha level for 13 CpGs)

A total of 10 associations were significant between the 13 CpG sites and the five TOD measures (FDR q < 0.1, Table 3): two CpGs were associated with eGFR (cg06690548 and cg10601624), six with UACR (cg16246545, cg14476101, cg19693031, cg06690548, cg00574958, and cg22304262), and two with LVMI (cg19693031 and cg00574958) before adjustment for BMI, smoking status and/or diabetes. When additionally adjusting for BMI or diabetes, both of the significant associations with eGFR, and four out of six significant associations with UACR remained significant (FDR q < 0.1), but both associations with LVMI were attenuated. After adjustment for smoking status, all associations remained significant (FDR q < 0.1).

**Table 3:** Associations between CpG sites and target organ damage in GENOA African Americans

CpG site	Gene	N	Unadjusted		BMI adjusted		Smoking adjusted		Diabetes adjusted		All adjusted	
			Beta	FDR q	Beta	FDR q	Beta	FDR q	Beta	FDR q	Beta	FDR q
***Estimated glomerular filtration rate***												
cg06690548	SLC7A11	1211	3.717	**0.004**	3.564	**0.004**	3.722	**0.004**	3.703	**0.004**	3.571	**0.004**
cg10601624	–	1211	−5.578	**0.005**	−5.661	**0.004**	−5.539	**0.005**	−5.625	**0.004**	−5.646	**0.004**
***Urine albumin-to-creatinine ratio***												
cg16246545	PHGDH	1213	−0.306	**0.066**	−0.279	0.106	−0.307	**0.066**	−0.194	0.356	−0.18	0.438
cg14476101	PHGDH	1213	−0.304	**0.006**	−0.274	**0.017**	−0.304	**0.006**	−0.213	**0.074**	−0.198	0.112
cg19693031	TXNIP	1213	−0.433	**1.3E-07**	−0.415	**4.6E-07**	−0.433	**1.3E-07**	−0.211	**0.024**	−0.212	**0.032**
cg06690548	SLC7A11	1213	−0.305	**0.001**	−0.296	**0.001**	−0.305	**0.001**	−0.264	**0.009**	−0.258	**0.012**
cg00574958	CPT1A	1213	−0.439	**5.9E-06**	−0.387	**1.1E-04**	−0.440	**5.8E-06**	−0.241	**0.024**	−0.218	**0.054**
cg22304262	SLC1A5	1213	−0.233	**0.087**	−0.222	0.108	−0.238	**0.084**	−0.146	0.383	−0.132	0.491
***Left ventricular mass / height^2.7***												
cg19693031	TXNIP	1170	−1.345	**0.058**	−0.743	0.601	−1.347	**0.057**	−0.342	0.883	−0.161	0.901
cg00574958	CPT1A	1170	−1.835	**0.031**	−0.663	0.601	−1.843	**0.030**	−1.116	0.678	−0.282	0.901

N: sample size, Beta: beta coefficient, SE: standard error, CpG: cytosine-phosphate-guanine, smoking: current smoking status

Gene names are from UCSC Genome Browser

All models were adjusted for age, sex, white blood cell counts, time difference between phase 1 and 2, and 10 genetic principal components

Urine albumin-to-creatinine ratio was log transformed

Only results with FDR q < 0.1 are shown in Table 3

FDR q-value was bolded if < 0.1

To evaluate whether there is an interaction between a CpG site and the traditional risk factors for arteriosclerosis (BMI, smoking status, and diabetes) on TOD measures, we added interaction terms in the baseline association models. We found four significant interactions on UACR: cg06690548-BMI, cg14476101-diabetes, cg19693031-diabetes, and cg06690548-diabetes (FDR q < 0.1, Table S1). The visualization of the significant interactions is shown in Figure S1. Increasing cg06690548 methylation was associated with a larger reduction in ln (UACR+ 1) for those with obesity (BMI ≥ 30) (Beta = − 0.59; 95% CI -8.83,-0.36) than those without obesity (Beta = − 0.02; 95% CI: − 0.24 to − 0.36). When stratifying by diabetes, the predicted value of UACR decreased as the level methylation of CpG sites (cg19693031, cg14476101, and cg06690548) increased (Beta = − 0.46, − 0.62, and − 0.54; 95% CI: − 0.76 to 0.16, − 1.02 to − 0.22, and − 0.86 to − 0.23, respectively) in those with diabetes, but the decrease in UACR was less pronounced in those without diabetes (Beta = − 0.05, − 0.05, and − 0.13; 95% CI: − 0.21 to 0.11, − 0.23 to 0.14, and − 0.29 to 0.04, respectively).

Using formal mediation analyses, we examined the indirect association between CpG sites and TOD measures (10 significant associations from the primary association analysis) through changes in blood pressure. Mediation analysis showed that seven associations between CpG sites and two TOD measures (UACR and LVMI) were significantly mediated by blood pressure (cg19693031 and cg00574958 by SBP for both UACR and LVMI, cg19693031 by DBP for UACR, cg19693031 and cg00574958 by DBP for UACR, proportion mediated: 4.7 to 15.7% for UACR and 14.5 to 38.1% for LVMI, Bonferroni-corrected *P* < 0.05, Table S2). However, the significance of three of the seven mediating effects by SBP were attenuated with further adjustment for BMI, smoking status, and diabetes in the mediation analysis (cg19693031 and cg00574958 by SBP for UACR, cg19693031 by SBP for LVMI) while all significant mediating effects by DBP remained (Table S3).

Using the 10 significant CpG site-TOD measure associations prior to adding arteriosclerosis risk factors (FDR q < 0.1), we conducted a 2-step association analysis to identify potential mediating effects of gene expression level: an association between each CpG site and expression of proximal genes, and an association between gene expression and the TOD measure. A total of five genes with expression data that were located within 1 Mb of a corresponding CpG site were at least marginally associated (*P* < 0.1) with both the corresponding CpG site and the TOD trait (cg06690548-*RP13-884E18.4*-eGFR, cg10601624-*CD4*-eGFR, cg00574958-*AP000439.3*-UACR, cg22304262-*FOXA3*-UACR, and cg22304262-*CCDC61*-UACR). We then performed a formal mediation analysis for these five associations but found no significant (*P* < 0.05) mediating effect of gene expression on the associations.

We conducted MR analysis in GENOA African American participants to provide support for causal relationships between DNA methylation and TOD. Specifically, we used inverse-variance weighted (IVW) tests to examine forward causality of DNA methylation on TOD. The results of MR analysis of CpG-TOD association are shown in the Table S4. In the absence of pleiotropic effects, IVW tests suggest that UACR is inversely influenced by DNA methylation at two CpG sites located within *PHGDH*, cg16246545 (Beta = − 0.50 and *P* = 1.5E-04) and cg14476101 (Beta = − 0.37 and *P* = 5.8E-06, Figure S2). The significant causal effect estimates are consistent in magnitude and direction with those estimated by our baseline association analysis between the CpG sites and UACR (Beta = − 0.306 for cg16246545 and Beta = − 0.304 for cg14476101). Instrumental variables were not available for cg06690548 and cg10601624. The MR Egger test suggested that there was no evidence of horizontal pleiotropic effects of the instrumental variables.

## Discussion

We identified ten significant associations between previously identified CpG sites associated with blood pressure and TOD measures including eGFR, UACR, and/or LVMI in a sample of 1218 African Americans. Half of these associations remained significant even after further adjustment for other TOD risk factors including BMI, smoking status, and diabetes. Among the CpG sites from the ten significant associations, one significant interaction with BMI and three significant interactions with diabetes on UACR were identified in an interaction analysis. Seven associations between CpG sites and two TOD measures were partially mediated by blood pressure (4.7 to 38.1%), but there was no significant *cis* gene expression mediating the associations between CpG sites and TOD measures. MR analysis provided supporting evidence of the forward direction of association for 2 CpG sites located near *PHGDH* with UACR.

Only a small portion of previously identified CpG sites from Richard et al. (2017) were also significantly associated with blood pressure in GENOA after multiple testing correction (three out of 13 CpG sites). Syme et al. (2019) found three out of the 13 CpG sites had significant associations with blood pressure in 135 adolescents and 110 middle-aged adults [16]. Most of the CpG sites in GENOA and Syme et al. (2019) had the same direction of effects as in Richard et al. (2017). However, none of CpG sites from two studies with significant association with blood pressure were overlapped (cg19693031, cg18120259, cg00574958 in GENOA and cg14476101, cg06690548, cg18120259 in Syme et al.). The low rate of replication might be due to the smaller sample size or the characteristics of subjects in the two studies.

Results from the mediation analysis suggests the significant associations between the CpG sites and TOD are not always mediated through blood pressure. Since the GENOA participants were selected based on their own or their sibling’s hypertension status, the average blood pressure in GENOA was elevated compared to the general population. Some of these CpG sites from Richard et al. (2017) may operate at a lower blood pressure level, but may have weaker effects at higher blood pressures, so associations may not be as pronounced in GENOA. Despite the lower rate of replication with blood pressure, we found 10 significant associations with TOD measures in the kidney and heart (eGFR, UACR, and LVMI).

We identified significant associations between cg10601624, cg06690548 (*SLC7A11*), cg19693031 (*TXNIP*), and cg00574958 (*CPT1A*) and TOD in the kidney even further after adjustment for BMI, smoking status, and diabetes. cg06690548 (*SLC7A11*), cg19693031 (*TXNIP*) and cg00574958 (*CPT1A*) have previously been associated with triglyceride levels, in addition to blood pressure, in EWAS studies [15, 33]. However, MR analyses for these two EWAS findings could not provide clear evidence for the direction of associations due to the lack of appropriate genetic instrument available in GENOA [15, 34]. In our MR analysis, we identified two CpG sites at *PHGDH* that may causally influence affect UACR. *PHGDH* encodes an enzyme which facilitates amino acid L-serine biosynthesis. Disturbances in serine metabolism are associated with endothelial dysfunction, inflammation, and atherosclerosis in the kidney, and there is evidence that the mechanism of these effects may be through the 1-carbon (DNA methylation) metabolism pathway [35, 36]. The decline in DNA methylation levels of cg14476101 has been also linked to high triglycerides levels [37], blood concentrations of 4-androstene-3beta,17beta-diol, a steroid hormone [38], and obesity [39], which play a role in the pathogenesis of atherosclerosis [40].

We evaluated whether the CpG sites were associated with TOD measures through transcriptional changes, but there was no significant gene expression mediating the associations between the CpG sites and TOD measures. In GENOA, the methylation and gene expression levels were measured in different tissues: methylation in peripheral blood leukocytes and gene expression in lymphoblastroid cell lines, which may be one possible reason for the lack of significant results. The DNA methylation profiles between peripheral blood leukocytes and lymphoblastroid cell lines are not always consistent [41]. In addition, we only examined gene expression located within ±1 Mb of the corresponding CpG sites, so we could have missed distal regulatory effects.

The inverse association between DNA methylation and UACR was stronger in those with diabetes (cg14476101 in *PHGDH*, cg19693031 in *TXNIP*, and cg06690548 in *SLC7A11*) or those who were obese (cg06690548 in *SLC7A11*). *PHGDH* is previously reported to be involved in the progression of glucose intolerance through serine biosynthesis in mice adipose tissues [42], while expression of *TXNIP* is increased in the presence of high glucose, which promotes oxidative stress and β-cell apoptosis [43]. We found no significant interaction between DNA methylation and smoking on TOD, but few GENOA participants reported that they currently smoked.

With multiple measures of TOD and omics data (epigenomic and transcriptomics), we ascertained the relationships between TOD measures and previously identified DNA methylation levels associated with blood pressure using state-of-the-art analytic methods in a population with a high burden of hypertension. In GENOA, TOD traits were measured approximately 5 years later than methylation, thus helping to establish the temporal relationship between changes in methylation and TOD. We were also able to characterize the direction of association using MR techniques. However, for two CpG sites (cg06690548 and cg10601624), we were not able to examine the causal direction of the association because of the lack of appropriate instruments. Also, despite of the five-year lag between measurements, the level of TOD trait could have been changed before methylation level changed. In addition, we lacked the ability to account for the duration of hypertension, which preceded DNA methylation for over two thirds of the study population. When we adjusted for hypertension in the baseline association models, the significance remained for eGFR and UACR, but attenuated in LVMI. Studies with longitudinal measures of DNA methylation at multiple timepoints may help better elucidate these temporal relationships.

## Conclusions

In conclusion, we found compelling evidence for associations between arteriosclerotic traits of kidney and heart and previously identified blood pressure-associated DNA methylation sites in a cohort of older African Americans. For the identified associations, we additionally examined the interactions between CpGs and other risk factors, potential mediation by blood pressure, and the causal relationship connecting CpG sites and TOD measures. We found evidence supporting the causal relationship of epigenetic regulation of *PHGDH* in the kidney, potentially through endothelial dysfunction, inflammation, and atherosclerosis. Future research should further confirm these initial findings in populations of other ancestries. This study may lend insight into the role of DNA methylation in complex pathological mechanisms underlying target organ damage from hypertension and provide potential clinical targets for treatment and prevention.

## Supplementary information

**Additional file 1: Table S1**. Interactions between CpG sites and target organ damage risk factors including BMI, current smoking, and diabetes on target organ damage phenotypes in GENOA African ancestry population. **Table S2**. Results of mediation analysis of CpG sites-systolic blood pressure-target organ damage phenotypes in GENOA African ancestry population. **Table S3**. Results of mediation analysis of CpG sites-systolic blood pressure-target organ damage phenotypes adjusted for BMI, current smoking, and diabetes in GENOA African ancestry population. **Table S4**. Mendelian randomization results showing the inverse-variance weighted effects of multiple cis-SNP used as instrumental variables in the association of DNA methylation and target organ damage measures in GENOA African ancestry population. **Figure S1**. Interaction plots between a CpG site and the traditional risk factors for arteriosclerosis (BMI and diabetes) on urine albumin-to-creatinine ratio (UACR). **Figure S2**. Mendelian randomization (MR) scatterplot of urine albumin-to-creatinine ratio vs. CpG with estimates from inverse-variance weighted (IVW) MR using cis-SNPs within ±1 Mb of corresponding CpG site in GENOA African Americans.

## Data Availability

For this analysis, genotype and phenotype data are from the Database of Genotypes and Phenotypes (dbGaP): phs001401.v2.p1. Gene expression and methylation data are from the Gene Expression Omnibus (GEO): GSE138914 and GSE157131, respectively. Due to IRB restriction, mapping of the sample IDs between genotype data (dbGaP) and gene expression data (GEO) cannot be provided publicly, but are available upon written request to JAS and SLRK.

## Abbreviations

ABI: Ankle-brachial index
BMI: Body mass index
BP: Blood pressure
DBP: Diastolic blood pressure
eGFR: Estimated glomerular filtration rate
EWAS: Epigenome-wide association studies
FDR: False discovery rate
GENOA: Genetic Epidemiology Network of Arteriopathy
IVW: Inverse-variance weighting
LVMI: Left ventricular mass indexed to height
MAF: Minor allele frequency
MR: Mendelian randomization
PCs: Principal components
RWT: Relative wall thickness
SBP: Systolic blood pressure
TOD: Target organ damage
UACR: Urinary albumin-to-creatinine ratio

## References

[1] Benjamin EJ, Virani SS, Callaway CW, Chamberlain AM, Chang AR, Cheng S (2018). Heart disease and stroke statistics—2018 update: a report from the American Heart Association. Circulation..

[2] MacMahon S, Peto R, Collins R, Godwin J, Cutler J, Sorlie P (1990). Blood pressure, stroke, and coronary heart disease: part 1, prolonged differences in blood pressure: prospective observational studies corrected for the regression dilution bias. Lancet.

[3] Cohuet G, Struijker-Boudier H (2006). Mechanisms of target organ damage caused by hypertension: therapeutic potential. Pharmacol Ther.

[4] Shlomai G, Grassi G, Grossman E, Mancia G (2013). Assessment of target organ damage in the evaluation and follow-up of hypertensive patients: where do we stand?. J Clin Hypertens.

[5] Harbaoui B, Courand P-Y, Defforges A, Khettab F, Milon H, Girerd N (2015). Cumulative effects of several target organ damages in risk assessment in hypertension. Am J Hypertens.

[6] Crews DC, Plantinga LC, Miller ER, Saran R, Hedgeman E, Saydah SH (2010). Prevalence of chronic kidney disease in persons with undiagnosed or prehypertension in the United States. Hypertension..

[7] Cuspidi C, Sala C, Negri F, Mancia G, Morganti A (2012). Prevalence of left-ventricular hypertrophy in hypertension: an updated review of echocardiographic studies. J Hum Hypertens.

[8] Levy D, Ehret GB, Rice K, Verwoert GC, Launer LJ, Dehghan A (2009). Genome-wide association study of blood pressure and hypertension. Nat Genet.

[9] Ehret GB, Munroe PB, Rice KM, Bochud M, Johnson AD, Chasman DI (2011). Genetic variants in novel pathways influence blood pressure and cardiovascular disease risk. Nature..

[10] Köttgen A, Pattaro C, Böger CA, Fuchsberger C, Olden M, Glazer NL (2010). New loci associated with kidney function and chronic kidney disease. Nat Genet.

[11] Wuttke M, Köttgen A (2016). Insights into kidney diseases from genome-wide association studies. Nat Rev Nephrol.

[12] Arnett DK, Meyers KJ, Devereux RB, Tiwari HK, Gu CC, Vaughan LK (2011). Genetic variation in NCAM1 contributes to left ventricular wall thickness in hypertensive families. Circ Res.

[13] Murabito JM, White CC, Kavousi M, Sun YV, Feitosa MF, Nambi V (2012). Association between chromosome 9p21 variants and the ankle-brachial index identified by a meta-analysis of 21 genome-wide association studies. Circ Cardiovasc Genet.

[14] Chu AY, Tin A, Schlosser P, Ko YA, Qiu C, Yao C (2017). Epigenome-wide association studies identify DNA methylation associated with kidney function. Nat Commun.

[15] Richard MA, Huan T, Ligthart S, Gondalia R, Jhun MA, Brody JA (2017). DNA methylation analysis identifies loci for blood pressure regulation. Am J Hum Genet.

[16] Syme C, Shin J, Richer L, Gaudet D, Fornage M, Paus T (2019). Epigenetic loci of blood pressure: underlying hemodynamics in adolescents and adults. Circulation: Genomic and Precision Medicine.

[17] Williams RR, Rao D, Ellison RC, Arnett DK, Heiss G, Oberman A (2000). NHLBI family blood pressure program: methodology and recruitment in the HyperGEN network. Ann Epidemiol.

[18] Family Blood Pressure Program Investigators. Multi-center genetic study of hypertension the family blood pressure program (FBPP). Hypertension. 2002;39(1):3–9. 10.1161/hy1201.100415 11799070

[19] Aryee MJ, Jaffe AE, Corrada-Bravo H, Ladd-Acosta C, Feinberg AP, Hansen KD (2014). Minfi: a flexible and comprehensive bioconductor package for the analysis of Infinium DNA methylation microarrays. Bioinformatics..

[20] Smith JA, Raisky J, Ratliff SM, Liu J, Kardia SLR, Turner ST (2019). Intrinsic and extrinsic epigenetic age acceleration are associated with hypertensive target organ damage in older African Americans. BMC Med Genet.

[21] Houseman EA, Accomando WP, Koestler DC, Christensen BC, Marsit CJ, Nelson HH (2012). DNA methylation arrays as surrogate measures of cell mixture distribution. BMC Bioinformatics.

[22] Du P, Zhang X, Huang C-C, Jafari N, Kibbe WA, Hou L (2010). Comparison of Beta-value and M-value methods for quantifying methylation levels by microarray analysis. BMC Bioinformatics.

[23] Johnson WE, Li C, Rabinovic A (2007). Adjusting batch effects in microarray expression data using empirical Bayes methods. Biostatistics..

[24] Irizarry RA, Bolstad BM, Collin F, Cope LM, Hobbs B, Speed TP (2003). Summaries of Affymetrix GeneChip probe level data. Nucleic Acids Res.

[25] Dai M, Wang P, Boyd AD, Kostov G, Athey B, Jones EG (2005). Evolving gene/transcript definitions significantly alter the interpretation of GeneChip data. Nucleic Acids Res.

[26] Tobin MD, Sheehan NA, Scurrah KJ, Burton PR (2005). Adjusting for treatment effects in studies of quantitative traits: antihypertensive therapy and systolic blood pressure. Stat Med.

[27] Team RC (2013). R: a language and environment for statistical computing.

[28] Tingley D, Yamamoto T, Hirose K, Keele L, Imai K (2014). Mediation: R package for causal mediation analysis.

[29] Zhan X, Hu Y, Li B, Abecasis GR, Liu DJ (2016). RVTESTS: an efficient and comprehensive tool for rare variant association analysis using sequence data. Bioinformatics..

[30] Johnson T, Uk S (2012). Efficient calculation for multi-SNP genetic risk scores.

[31] Hemani G, Zheng J, Elsworth B, Wade KH, Haberland V, Baird D, et al. The MR-base platform supports systematic causal inference across the human phenome. Elife. 2018;7:e34408. 10.7554/eLife.34408 PMC5976434 29846171

[32] Hemani G, Bowden J, Davey SG (2018). Evaluating the potential role of pleiotropy in Mendelian randomization studies. Hum Mol Genet.

[33] Sayols-Baixeras S, Subirana I, Lluis-Ganella C, Civeira F, Roquer J, Do AN (2016). Identification and validation of seven new loci showing differential DNA methylation related to serum lipid profile: an epigenome-wide approach. The REGICOR study. Hum Mol Genet.

[34] Sayols-Baixeras S, Tiwari HK, Aslibekyan SW (2018). Disentangling associations between DNA methylation and blood lipids: a Mendelian randomization approach. BMC Proc.

[35] Ovrehus MA, Bruheim P, Ju W, Zelnick LR, Langlo KA, Sharma K (2019). Gene expression studies and targeted metabolomics reveal disturbed serine, methionine, and tyrosine metabolism in early hypertensive Nephrosclerosis. Kidney Int Rep.

[36] Rodriguez AE, Ducker GS, Billingham LK, Martinez CA, Mainolfi N, Suri V (2019). Serine Metabolism Supports Macrophage IL-1beta Production. Cell Metab.

[37] Truong V, Huang S, Dennis J, Lemire M, Zwingerman N, Aissi D (2017). Blood triglyceride levels are associated with DNA methylation at the serine metabolism gene PHGDH. Sci Rep.

[38] Petersen AK, Zeilinger S, Kastenmuller G, Romisch-Margl W, Brugger M, Peters A (2014). Epigenetics meets metabolomics: an epigenome-wide association study with blood serum metabolic traits. Hum Mol Genet.

[39] Aslibekyan S, Demerath EW, Mendelson M, Zhi D, Guan W, Liang L (2015). Epigenome-wide study identifies novel methylation loci associated with body mass index and waist circumference. Obesity (Silver Spring).

[40] Talayero BG, Sacks FM (2011). The role of triglycerides in atherosclerosis. Curr Cardiol Rep.

[41] Lokk K, Modhukur V, Rajashekar B, Martens K, Magi R, Kolde R (2014). DNA methylome profiling of human tissues identifies global and tissue-specific methylation patterns. Genome Biol.

[42] Okabe K, Usui I, Yaku K, Hirabayashi Y, Tobe K, Nakagawa T (2018). Deletion of PHGDH in adipocytes improves glucose intolerance in diet-induced obese mice. Biochem Biophys Res Commun.

[43] Thielen L, Shalev A (2018). Diabetes pathogenic mechanisms and potential new therapies based upon a novel target called TXNIP. Curr Opin Endocrinol Diabetes Obes.

