# Supplementary material for: Epigenetic loci for blood pressure are associated with hypertensive target organ damage in older African Americans from the genetic epidemiology network of Arteriopathy (GENOA) study
Source: BMC Med Genomics. 2020 Sep 11;13:131. doi: 10.1186/s12920-020-00791-0 (PMC7488710; doi:10.1186/s12920-020-00791-0)

Table S1. Interactions between CpG sites and target organ damage risk factors including BMI, current smoking, and diabetes on target organ damage phenotypes in GENOA African ancestry population

		CpG x BMI			CpG x Current smoking				CpG x Diabetes		
CpG site	N	Beta	SE	FDR q	N	Beta	SE	FDR q	N	Beta	SE	FDR q
Estimated glomerular filtration rate												
cg06690548	1,207	0.147	0.141	0.473	1,211	-2.901	2.882	0.559	1,211	-0.975	2.103	0.857
cg10601624	1,207	0.273	0.244	0.469	1,211	-6.420	5.143	0.446	1,211	-1.963	3.572	0.847
Urine albumin-to-creatinine ratio												
cg16246545	1,208	-0.011	0.020	0.722	1,213	0.184	0.392	0.729	1,209	-0.207	0.272	0.847
cg14476101	1,208	-0.016	0.015	0.469	1,213	-0.017	0.275	0.950	1,209	-0.462	0.192	**0.087**
cg19693031	1,208	-0.015	0.010	0.383	1,213	0.322	0.207	0.410	1,209	-0.459	0.149	**0.034**
cg06690548	1,208	-0.029	0.011	**0.073**	1,213	0.328	0.230	0.410	1,209	-0.436	0.159	**0.049**
cg00574958	1,208	0.001	0.013	0.953	1,213	0.305	0.250	0.446	1,209	0.021	0.184	0.943
cg22304262	1,208	-0.010	0.016	0.722	1,213	0.177	0.307	0.729	1,209	-0.386	0.230	0.267
Left ventricular mass / height^2.7												
cg19693031	1,169	-0.109	0.069	0.372	1,170	-0.443	1.388	0.800	1,166	-1.961	1.055	0.253
cg00574958	1,169	-0.128	0.080	0.372	1,170	3.153	1.653	0.377	1,166	0.279	1.286	0.943

N: sample size; Beta: coefficient; SE: standard error; CpG: cytosine-phosphate-guanine; smoking: current smoking status
All models were adjusted for age, sex, blood cell counts, time difference between phase 1 and 2, and 10 principal components.
Urine albumin-to-creatinine ratio was log transformed.
FDR q value was bolded if less than 0.1

Table S2. Results of mediation analysis of CpG sites-systolic blood pressure-target organ damage phenotypes in GENOA African ancestry population

		Total effect			Direct effect			Mediation effect			Proportion mediated		
CpG	N	B	BF P	95% CI	B	BF P	95% CI	B	BF P	95% CI	PM	BF P	95% CI
CpG site-systolic blood pressure-target organ damage													
Estimated glomerular filtration rate													
cg06690548	1212	3.47	**0.0014**	(1.49 , 5.45)	3.49	**0.0012**	(1.50 , 5.46)	-0.022	0.856	(-0.27 , 0.22)	-0.006	0.8574	(-0.10 , 0.07)
cg10601624	1212	-5.37	**0.0012**	(-8.59 , -2.15)	-5.32	**0.0016**	(-8.53 , -2.14)	-0.052	0.782	(-0.45 , 0.33)	0.009	0.7816	(-0.08 , 0.10)
Urine albumin-to-creatinine ratio													
cg16246545	1214	-0.32	0.0214	(-0.58 , -0.04)	-0.31	0.0204	(-0.57 , -0.05)	-0.004	0.8828	(-0.06 , 0.06)	0.014	0.8758	(-0.37 , 0.28)
cg14476101	1214	-0.31	**0.0018**	(-0.50 , -0.12)	-0.29	**0.0016**	(-0.47 , -0.10)	-0.019	0.3688	(-0.06 , 0.02)	0.060	0.3686	(-0.10 , 0.24)
cg19693031	1214	-0.43	**0**	(-0.58 , -0.29)	-0.39	**0**	(-0.53 , -0.25)	-0.042	**0.0082**	(-0.08 , -0.01)	0.095	**0.0082**	(0.03 , 0.19)
cg06690548	1214	-0.30	**0**	(-0.46 , -0.14)	-0.30	**0**	(-0.46 , -0.15)	0.004	0.8262	(-0.03 , 0.04)	-0.013	0.8262	(-0.18 , 0.11)
cg00574958	1214	-0.42	**0**	(-0.59 , -0.25)	-0.36	**2.00E-04**	(-0.52 , -0.19)	-0.067	**6.00E-04**	(-0.11 , -0.03)	0.157	**6.00E-04**	(0.07 , 0.30)
cg22304262	1214	-0.25	0.0316	(-0.47 , -0.02)	-0.22	0.049	(-0.44 , 0.00)	-0.027	0.2954	(-0.08 , 0.02)	0.106	0.3042	(-0.18 , 0.61)
Left ventricular mass / height^2.7													
cg19693031	1171	-1.24	**0.0168**	(-2.24 , -0.22)	-0.78	0.1084	(-1.73 , 0.18)	-0.459	**0.0052**	(-0.79 , -0.14)	0.366	**0.0208**	(0.10 , 1.35)
cg00574958	1171	-1.75	**0.0036**	(-2.89 , -0.58)	-1.08	0.059	(-2.17 , 0.04)	-0.668	**4.00E-04**	(-1.07 , -0.29)	0.381	**0.004**	(0.17 , 1.04)
CpG site-diastolic blood pressure-target organ damage													
Estimated glomerular filtration rate													
cg06690548	1212	3.48	**0.0014**	(1.49 , 5.47)	3.45	**0.0012**	(1.46 , 5.45)	0.022	0.8014	(-0.18 , 0.23)	0.006	0.8004	(-0.06 , 0.08)
cg10601624	1212	-5.37	**0.0014**	(-8.59 , -2.14)	-5.38	**0.0014**	(-8.58 , -2.17)	0.011	0.938	(-0.32 , 0.34)	-0.002	0.9382	(-0.08 , 0.07)
Urine albumin-to-creatinine ratio													
cg16246545	1214	-0.32	0.0198	(-0.59 , -0.05)	-0.33	0.0164	(-0.60 , -0.06)	0.008	0.5482	(-0.02 , 0.04)	-0.021	0.5608	(-0.25 , 0.08)
cg14476101	1214	-0.31	**0.001**	(-0.50 , -0.12)	-0.31	**0.001**	(-0.50 , -0.12)	-0.001	0.884	(-0.02 , 0.02)	0.004	0.8838	(-0.08 , 0.08)
cg19693031	1214	-0.43	**0**	(-0.58 , -0.29)	-0.41	**0**	(-0.56 , -0.27)	-0.022	**0.004**	(-0.04 , -0.01)	0.049	**0.004**	(0.01 , 0.11)
cg06690548	1214	-0.30	**0**	(-0.46 , -0.15)	-0.30	**2.00E-04**	(-0.46 , -0.14)	-0.002	0.7906	(-0.02 , 0.01)	0.006	0.7906	(-0.06 , 0.07)
cg00574958	1214	-0.42	**0**	(-0.59 , -0.25)	-0.40	**2.00E-04**	(-0.57 , -0.23)	-0.021	0.01	(-0.05 , 0.00)	0.047	0.01	(0.01 , 0.12)
cg22304262	1214	-0.25	0.0308	(-0.47 , -0.02)	-0.23	0.0436	(-0.45 , -0.01)	-0.018	0.1008	(-0.05 , 0.00)	0.065	0.1272	(-0.03 , 0.41)
Left ventricular mass / height^2.7													
cg19693031	1171	-1.26	**0.0144**	(-2.26 , -0.25)	-0.97	0.0598	(-1.95 , 0.05)	-0.296	**4.00E-04**	(-0.50 , -0.12)	0.232	**0.0144**	(0.08 , 0.94)
cg00574958	1171	-1.76	**0.003**	(-2.92 , -0.59)	-1.50	**0.0114**	(-2.64 , -0.34)	-0.259	**0.005**	(-0.49 , -0.07)	0.145	**0.008**	(0.04 , 0.45)

N: sample size; Beta: coefficient; CpG: cytosine-phosphate-guanine; smoking: current smoking status, CI: confidence interval, PM: proportion mediated

All models were adjusted for age and sex

Urine albumin-to-creatinine ratio was log transformed

BF indicates Bonferroni-corrected P value and cell was colored if <0.05/number of tests per each target organ measure

Table S3. Results of mediation analysis of CpG sites-systolic blood pressure-target organ damage phenotypes adjusted for BMI, current smoking, and diabetes in GENOA African ancestry population

		Total effect			Direct effect			Mediation effect			Proportion mediated		
CpG	N	B	BF P	95% CI	B	BF P	95% CI	B	BF P	95% CI	PM	BF P	95% CI
CpG site-systolic blood pressure-target organ damage													
Estimated glomerular filtration rate													
cg06690548	1208	3.32	**0.001**	(1.32 , 5.30)	3.37	**8.00E-04**	(1.38 , 5.37)	-0.054	0.646	(-0.31 , 0.18)	-0.015	0.6458	(-0.13 , 0.06)
cg10601624	1208	-5.44	**8.00E-04**	(-8.63 , -2.26)	-5.32	**8.00E-04**	(-8.48 , -2.13)	-0.124	0.515	(-0.55 , 0.25)	0.021	0.5158	(-0.06 , 0.12)
Urine albumin-to-creatinine ratio													
cg16246545	1205	-0.20	0.1354	(-0.46 , 0.06)	-0.20	0.111	(-0.46 , 0.05)	0.006	0.793	(-0.04 , 0.06)	-0.019	0.862	(-1.05 , 0.87)
cg14476101	1205	-0.20	0.0332	(-0.39 , -0.02)	-0.20	0.0368	(-0.38 , -0.01)	-0.008	0.661	(-0.04 , 0.03)	0.035	0.6638	(-0.28 , 0.34)
cg19693031	1205	-0.22	**0.004**	(-0.36 , -0.07)	-0.20	**0.007**	(-0.34 , -0.05)	-0.017	0.215	(-0.05 , 0.01)	0.078	0.2162	(-0.06 , 0.29)
cg06690548	1205	-0.25	**0.0016**	(-0.40 , -0.10)	-0.26	**8.00E-04**	(-0.41 , -0.11)	0.007	0.6364	(-0.02 , 0.04)	-0.028	0.638	(-0.22 , 0.09)
cg00574958	1205	-0.20	0.0242	(-0.37 , -0.03)	-0.16	0.0626	(-0.33 , 0.01)	-0.038	0.0172	(-0.07 , -0.01)	0.185	0.041	(0.01 , 0.87)
cg22304262	1205	-0.15	0.1814	(-0.36 , 0.07)	-0.13	0.2122	(-0.34 , 0.08)	-0.011	0.5768	(-0.05 , 0.03)	0.067	0.6126	(-0.82 , 1.10)
Left ventricular mass / height^2.7													
cg19693031	1166	-0.10	0.8452	(-1.08 , 0.88)	0.11	0.8164	(-0.81 , 1.05)	-0.206	0.1662	(-0.51 , 0.08)	0.221	0.7922	(-6.63 , 5.87)
cg00574958	1166	-0.28	0.626	(-1.41 , 0.84)	0.11	0.8386	(-0.96 , 1.18)	-0.390	**0.0222**	(-0.75 , -0.06)	0.495	0.6226	(-9.64 , 10.67)
CpG site-diastolic blood pressure-target organ damage													
Estimated glomerular filtration rate													
cg06690548	1208	3.33	**8.00E-04**	(1.32 , 5.32)	3.31	**0.0012**	(1.31 , 5.30)	0.017	0.8698	(-0.18 , 0.23)	0.004	0.8702	(-0.07 , 0.08)
cg10601624	1208	-5.43	**6.00E-04**	(-8.62 , -2.24)	-5.42	**6.00E-04**	(-8.60 , -2.24)	-0.008	0.9694	(-0.35 , 0.32)	0.001	0.9688	(-0.08 , 0.07)
Urine albumin-to-creatinine ratio													
cg16246545	1205	-0.20	0.1274	(-0.46 , 0.05)	-0.21	0.109	(-0.47 , 0.05)	0.008	0.4838	(-0.02 , 0.04)	-0.030	0.5608	(-0.59 , 0.43)
cg14476101	1205	-0.21	0.0326	(-0.39 , -0.02)	-0.20	0.0318	(-0.39 , -0.02)	0.000	0.9776	(-0.02 , 0.02)	0.001	0.9734	(-0.16 , 0.15)
cg19693031	1205	-0.22	**0.0036**	(-0.36 , -0.07)	-0.20	0.0094	(-0.34 , -0.05)	-0.020	**0.005**	(-0.04 , 0.00)	0.091	0.0086	(0.02 , 0.31)
cg06690548	1205	-0.25	**0.001**	(-0.40 , -0.10)	-0.25	**0.0014**	(-0.40 , -0.10)	-0.001	0.8874	(-0.02 , 0.01)	0.003	0.888	(-0.07 , 0.07)
cg00574958	1205	-0.20	0.0238	(-0.37 , -0.03)	-0.18	0.037	(-0.35 , -0.01)	-0.019	0.0118	(-0.04 , 0.00)	0.089	0.0352	(0.01 , 0.45)
cg22304262	1205	-0.15	0.1816	(-0.36 , 0.07)	-0.13	0.2328	(-0.34 , 0.08)	-0.015	0.1148	(-0.04 , 0.00)	0.079	0.2692	(-0.70 , 1.05)
Left ventricular mass / height^2.7													
cg19693031	1166	-0.13	0.7956	(-1.10 , 0.86)	0.16	0.7514	(-0.80 , 1.14)	-0.282	**4.00E-04**	(-0.49 , -0.11)	0.359	0.7952	(-8.27 , 8.79)
cg00574958	1166	-0.27	0.6326	(-1.40 , 0.85)	-0.04	0.9424	(-1.15 , 1.07)	-0.236	**0.0126**	(-0.46 , -0.05)	0.281	0.6336	(-6.42 , 6.30)

N: sample size; Beta: coefficient; CpG: cytosine-phosphate-guanine; Smoking: current smoking status, CI: confidence interval, PM: proportion mediated

All models were adjusted for age, sex, BMI, current smoking, and diabetes.

Urine albumin-to-creatinine ratio was log transformed.

BF indicates Bonferroni-corrected P value and cell was colored if <0.05/number of tests per each target organ measure

Table S4. Mendelian randomization results showing the inverse-variance weighted effects of multiple cis-SNP used as instrumental variables in the association of DNA methylation and target organ damage measures in GENOA African ancestry population

CpG	IV SNPs, n	Beta	SE	P value
Estimated glomerular filtration rate				
cg06690548	0	-	-	-
cg10601624	0	-	-	-
Urine albumin-to-creatinine ratio				
cg16246545	62	-0.504	0.133	**1.48E-04**
cg14476101	76	-0.369	0.081	**5.81E-06**
cg19693031	5	0.301	0.333	0.365
cg06690548	0	-	-	-
cg00574958 *	1	1.117	0.745	0.134
cg22304262	13	-0.054	0.273	0.842
Left ventricular mass / height^2.7				
cg19693031	5	1.404	2.283	0.539
cg00574958 *	1	2.839	5.029	0.572

IV SNPs, n: number of SNPs used for genetic instrumental variables; sample size; Beta: beta coefficient; SE: standard error; CpG: cytosine-phosphate-guanine;* Wald-ratio MR method was used if the number of IV SNPs =1;Urine albumin-to-creatinine ratio was log transformed; The beta coefficient is the pooled causal estimate from the Mendelian randomization analyses and should be interpreted as the effect per 1 change in DNA methylation (M-value) using genetic variants on target organ damage measure; The intercept term in MR Egger regression analysis did not show evidence for directional horizontal pleiotropy

Figure S1. Interaction plots between a CpG site and the traditional risk factors for arteriosclerosis (BMI and diabetes) on urine albumin-to-creatinine ratio (UACR).

UACR was log transformed prior to analysis. Participants were categorized based on their BMI (Non-obese group: BMI<30; obese group: BMI≥30) Only results with FDR q<0.1 are shown in Figure S1.

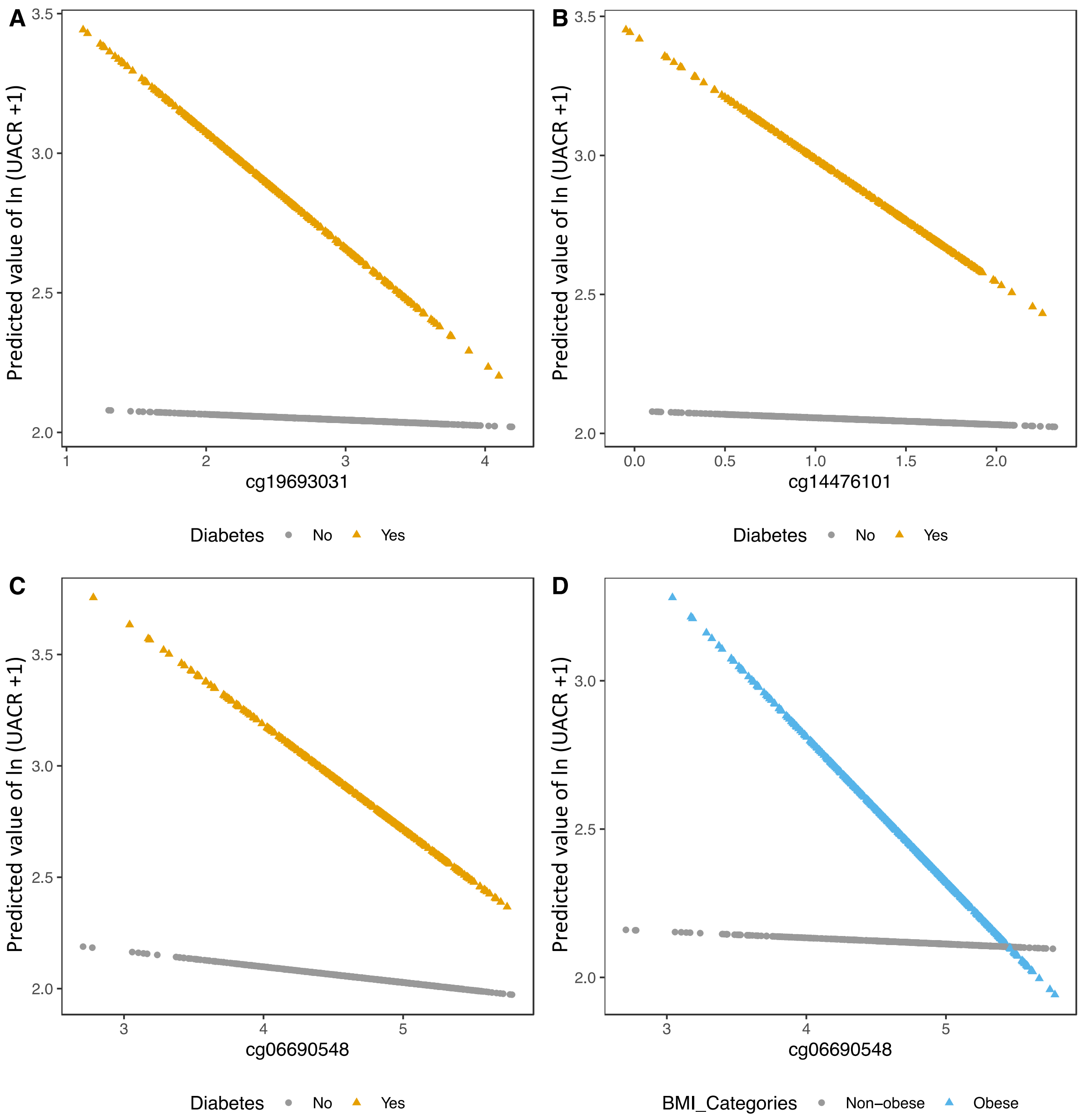

Figure S2. Mendelian randomization (MR) scatterplot of urine albumin-to-creatinine ratio vs. CpG with estimates from inverse-variance weighted (IVW) MR using cis-SNPs within ±1 Mb of corresponding CpG site in GENOA African Americans.

Lines identify the slopes of the IVW method (Beta=-0.50 and P= 1.48E-04 for cg16246545; Beta=-0.37 and P=5.81E-06 for cg14476101). Error bars represent standard errors of effect sizes.

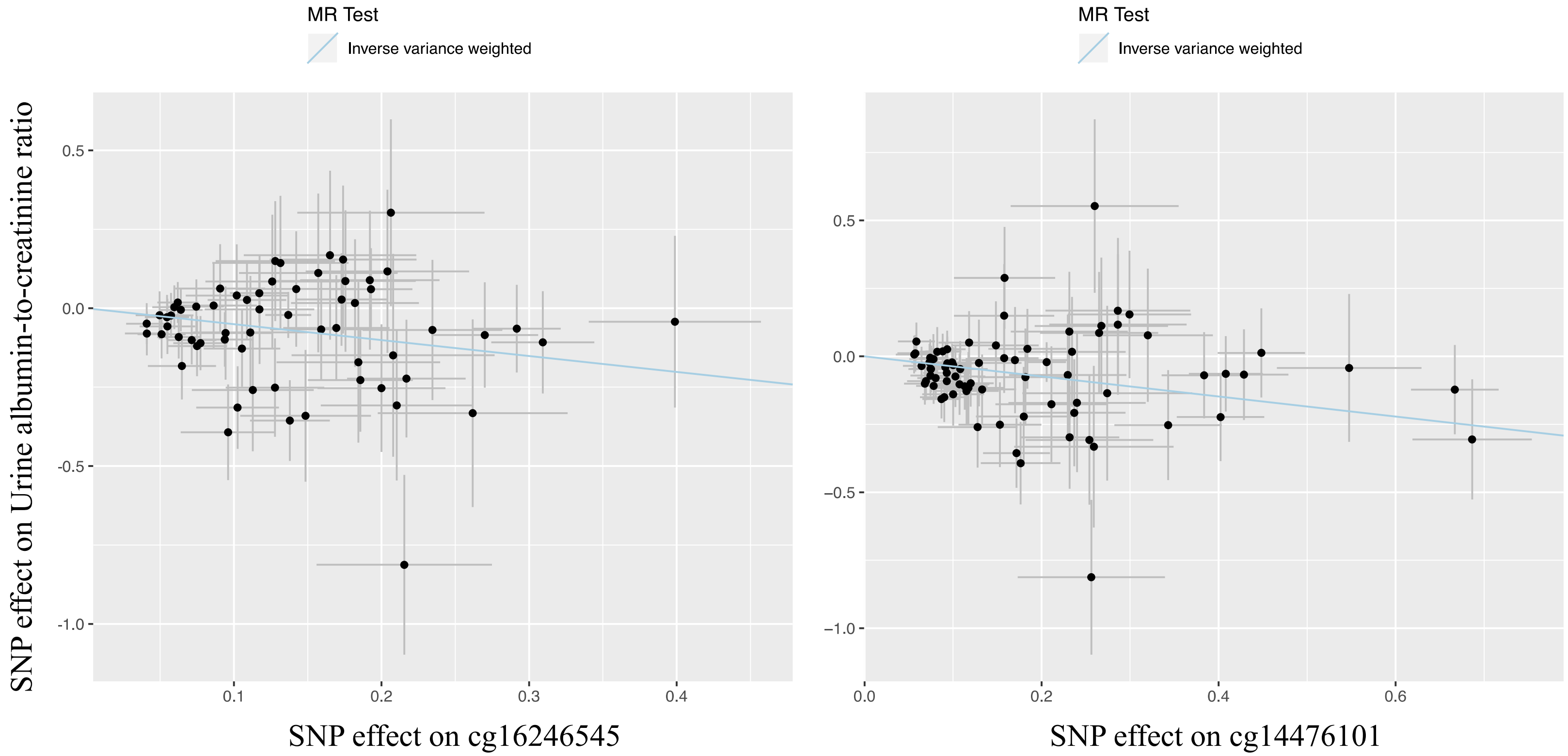

Supplement: Supplementary file 1 — Additional file 1: Table S1. Interactions between CpG sites and target organ damage risk factors including BMI, current smoking, and diabetes on target organ damage phenotypes in GENOA African ancestry population. Table S2. Results of mediation analysis of CpG sites-systolic blood pressure-target organ damage phenotypes in GENOA African ancestry population. Table S3. Results of mediation analysis of CpG sites-systolic blood pressure-target organ damage phenotypes adjusted for BMI, current smoking, and diabetes in GENOA African ancestry population. Table S4. Mendelian randomization results showing the inverse-variance weighted effects of multiple cis-SNP used as instrumental variables in the association of DNA methylation and target organ damage measures in GENOA African ancestry population. Figure S1. Interaction plots between a CpG site and the traditional risk factors for arteriosclerosis (BMI and diabetes) on urine albumin-to-creatinine ratio (UACR). Figure S2. Mendelian randomization (MR) scatterplot of urine albumin-to-creatinine ratio vs. CpG with estimates from inverse-variance weighted (IVW) MR using cis-SNPs within ±1 Mb of corresponding CpG site in GENOA African Americans. [file 12920_2020_791_MOESM1_ESM.pdf]
